# The dual role of the DREAM/G2M pathway in non‐tumorigenic immortalization of senescent cells

**DOI:** 10.1002/2211-5463.13748

**Published:** 2023-12-21

**Authors:** Jie Tian, Liangxia Jiang, Haili Li, Juhua Dan, Ying Luo

**Affiliations:** ^1^ Department of Pathophysiology, School of Basic Medicine Guizhou Medical University Guiyang China; ^2^ School of Basic Medicine Shandong First Medical University & Shandong Academy of Medical Sciences Jinan China; ^3^ Laboratory of Molecular Genetics of Aging & Tumor, Medical School Kunming University of Science and Technology China

**Keywords:** cell senescence, DREAM pathway, immortalization, tumorigenesis, Werner syndrome

## Abstract

Anti‐aging and tumorigenesis share common genes and pathways, and thus targeting these genes as part of anti‐aging interventions carries the risk of tumorigenesis. It is essential to understand the gene signatures that balance tumorigenesis and aging. To achieve this goal, we analyzed RNA‐sequencing data from three non‐tumorigenic immortalized cell lines that spontaneously escaped from senescence. By single sample gene set enrichment assay (ssGSEA) and GSEA analysis, we found that both cell growth signaling (E2F targets, MYC targets) and tumor surveillance mechanisms (DNA repair, G2M checkpoint, mitotic spindle) were up‐regulated in all three cell lines, suggesting that these genes are potential signatures for non‐tumorigenic immortalization. Further analysis revealed that the 182 commonly up‐regulated genes in these three cell lines overlapped with the DREAM/G2M pathway, which is known to be the upstream regulator of E2F, Myc targets, DNA repair, G2M checkpoint and mitotic spindle pathways in its cell cycle activation or inhibitory form. By western blotting, quantitative PCR and co‐immunoprecipitation, we verified that both forms of the DREAM pathway are up‐regulated in all three cell lines; this pathway facilitates control of cell cycle progression, supporting a new mechanism for non‐tumorigenic immortalization. Thus, we propose that the DREAM/G2M pathway plays important dual roles with respect to preventing tumorigenesis in the process of immortalization. Our data might serve as the basis for the identification of new signature pathways or gene biomarkers for non‐tumorigenic immortalization, and may aid in the discovery of new targets for tumor‐free anti‐aging drug screening.

## Graphical abstract

By analyzing the gene signatures from three independent non‐tumorigenic immortalized cell lines that spontaneously escaped from senescence, we reveal that the DREAM/G2M pathway plays important dual roles with respect to preventing tumorigenesis in the process of immortalization. Our data support a new mechanism for non‐tumorigenic immortalization, and may aid in the discovery of new targets for tumor‐free anti‐aging drug screening.

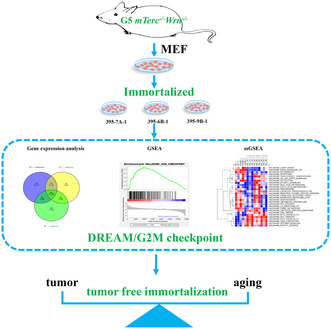

**Abbreviations**
G5DKO: fifth generation of WS MEFs
GSEA: gene set enrichment analysis
MEFs: mouse embryo fibroblasts
RNA‐seq: RNA‐sequencing
ssGSEA: single sample gene set enrichment analysis
WS: Werner syndrome
WT: wild‐type

Aging and its related diseases have become one of the most important fields of research. Artificial intervention of the aging process might prevent the incidence of aging‐related diseases. For this purpose, many studies have attempted to understand the genes or biological function signatures for longevity, either from the angle of mammalian species evolution or from the data derived from healthy aging population, such as centenarians.

A previous study was performed in primary skin fibroblasts isolated from 16 species of mammals aiming to identify genes and metabolites correlating with species longevity [1]. It was found that the DNA repair and glucose metabolism were up‐regulated in cells from longer lived species, whereas proteolysis and protein transport were down‐regulated. A single‐cell transcriptomic atlas across the lifespan of mice was generated to assess whether the most important hallmarks of aging, such as cell senescence, genomic instability and changes in the immune system, are reflected in a broad range of tissues and cell types. The data showed that the Cdkn2a had the highest correlation for cellular senescence, as well as E2f2, Lmnb1, Tnf and Itgax. For genes of the Sirt family (Sirt3, Sirt4 and Sirt5), the cells in which they were expressed were found to decrease with age [2].

The data from human population is complicated. Whole‐genome sequencing was performed for the Wellderly cohort, which included healthy individuals aged > 80 years [3]. Surprisingly, no major singular contributor to healthy aging was identified. Instead, healthy aging appears to demonstrate characteristics similar to complex polygenic phenotypes. RNA‐sequencing (RNA‐seq) has revealed that the autophagy‐lysosomal pathway is significantly up‐regulated in the blood cells of centenarians, indicating that clearance of senescent cells by autophagy‐lysosomal pathway is important for healthy aging [4]. A meta‐analysis of age‐related gene expression profiles using 27 datasets from mice, rats and humans revealed several common signatures of aging, including 56 genes consistently overexpressed with age, the most significant of which was APOD, and 17 genes underexpressed with age [5]. The age‐related biological processes involved an overexpression of inflammation and immune response genes and of genes associated with the lysosome. An underexpression of collagen genes and of genes associated with energy metabolism, particularly mitochondrial genes, as well as alterations in the expression of genes related to apoptosis, cell cycle and cellular senescence biomarkers, were also observed [5].

The above aging process related gene signatures provided us with markers and treatment targets for aging prevention. However, it is difficult to study a healthy population with the aim of providing a clear view with respect to tumor inhibition gene signatures along with the aging process. On the other hand, progeroid syndromes provided us with good model systems for investigating the genetic signatures of aging, as well as tumor prevention strategies. Werner syndrome (WS) is a rare autosomal recessive genetic disease. WS is caused by the mutation of RecQ family member Wrn, together with the developmental loss of telomerase activity in human somatic cells, manifested as Werner syndrome. The premature aging phenotypes of WS include a short average lifespan (46–48 years), early onset atherosclerosis, cataracts, osteoporosis, type II diabetes mellitus and an elevated incidence of soft tissue sarcoma [6]. After discovering the essential role of telomere dysfunction in the pathogenesis of WS, a mouse model of WS was generated by doubly knocking out Wrn and Terc (the RNA template of telomerase) [7]. The WS mouse model provides us a perfect accelerated aging model for interference of aging and its related diseases. However, as in regular human aging process, the control of the risk of tumorigenesis is essential in artificial interference.

It was also found that senescent WS mouse embryo fibroblasts (MEFs) could spontaneously escape from senescence and become immortalized [8]. Further study revealed that some of the immortalized cell lines (395‐3B‐1, 395‐3B‐2 and 395‐9B‐1) gain the p53N236S mutation (referred to as p53S) and methylation of the Ink4a locus. At the late stage, these cells are tumorigenic [9]. Some cell lines (395‐6B‐1, 395‐7A‐1) maintain the wild‐type (WT) status of p53, but spontaneously delete the Ink4a locus. These cells are not tumorigenic even at the late stage [10]. However, if we knock down p21 from these cells, 395‐6B‐1 becomes tumorigenic [11]. These cells provided us a perfect model for understanding the balance between aging and tumorigenesis. In the present study, we carried out RNA sequencing of 395‐7A‐1, 395‐6B‐1 and the early stage of 395‐9B‐1 cells. The control cells were their parental fifth generation of WS MEFs (G5 double mutation, G5DKO), as well as the WT MEFs. We analyzed the pathways regulated by immortalization and aimed to understand the key signature pathways or gene biomarkers that maintain cells non‐tumorigenic after immortalization. Our data revealed the dual role of the DREAM/G2M pathway with respect to preventing tumorigenesis in the process of promoting immortalization.

## Results

### Gene set enrichment assay (GSEA) analysis of pathways regulated by immortalization from senescent WS MEFs

The three independent lines of immortalized cells, 395‐7A‐1, 395‐6B‐1 and 395‐9B‐1, were spontaneously immortalized from senescent WS MEFs as previously described [8, 10]. The 395‐7A‐1, 395‐6B‐1 and 395‐9B‐1 cells were used for RNA‐seq, and the WT MEFs (WT) and fifth generation WS MEFs (G5DKO) were used as controls. The 395‐7A‐1, 395‐6B‐1 maintained WT p53 and could not form tumor in SCID mice [10]. However, the 395‐9B‐1 gained the p53S mutation and has potential to form tumor at its late stage [9]. In this work, we used the early stage of 395‐9B‐1, in which the p21 is still expressing, and the cells are non‐tumorigenic. When we knock down p21 in both 395‐7A‐1 and 395‐6B‐1 cells, only 395‐6B‐1 could form tumors with alternative lengthening of telomere mechanism [11]. Thus, the tumor potential for these three cell lines was 395‐7A‐1 < 395‐6B‐1 < 395‐9B‐1. These cell lines provided us with a gradual increase of tumor risk with which to evaluate the key pathways balancing tumorigenesis and aging.

**Fig. 1:**
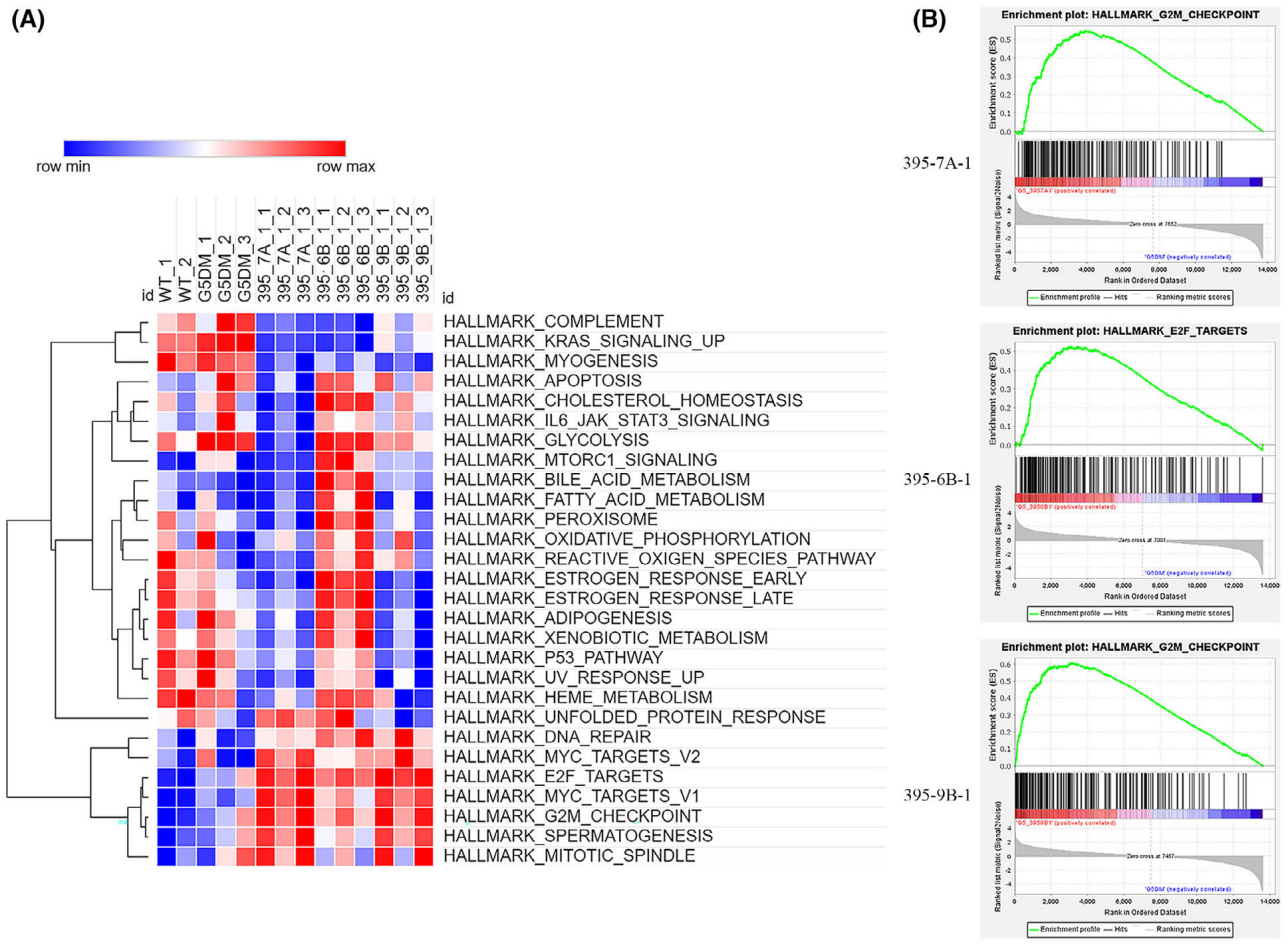
ssGSEA and GSEA analysis revealed the pathways regulated in 395‐7A‐1, 395‐6B‐1 and 395‐9B‐1 cells during immortalization (The Hallmark gene set was used as the pathway database). (A) Heatmaps plotted with ssGSEA score: red stands for up‐regulated pathways; blue stands for down‐regulated pathways. The WT MEFs and G5 generation WS MEFs (G5 double mutation, G5DKO) were used as controls. (B) The top 1 pathway in Hallmark gene set enriched by GSEA in 395‐7A‐1, 395‐6B‐1 and 395‐9B‐1 cells compared to their parental G5DKO cells.

The RNA‐seq data were normalized and analyzed by both single sample gene set enrichment assay (ssGSEA) and GSEA using the HALLMARK gene set, comprising coherently expressed signatures derived by aggregating many MSigDB gene sets to represent well‐defined biological states or processes [12]. To compare the expression profile changes in different group of samples, we first carried out the ssGSEA analysis. The up‐regulated or down‐regulated pathways were plotted with a heatmap. We found that compared with WT MEFs and their parental fifth generation of WS MEFs (G5DKO), the down‐regulated pathways in all three cell lines included COMPLEMENT, KRAS_SIGNALING_UP and MYOGENESIS. The up‐regulated pathways in all three cell lines included DNA_REPAIR, MYC_TARGETS, E2F_TARGETS, G2M_CHECKPOINT, SPERMATOGENESIS and MITOTIC_SPINDLE (Fig. 1A).

We then carried out the GSEA analysis to further confirm the ssGSEA results and dissect the pathways that are regulated in each immortalized cell line. Interestingly, we found that the top 1 enrichment pathway in 395‐7A‐1 and 395‐9B‐1 was G2M_CHECKPOINT, whereas the top 1 enrichment pathway in 395‐6B‐1 was E2F_TARGETS (Fig. 1B; the statistical data were presented in Table S1). Also, the results indicated that the common up‐regulated pathways in all three cell lines were G2M_CHECKPOINT and E2F_TARGETS. The MYC_TARGETS pathway was enriched in 395‐7A‐1 and 395‐9B‐1 cells. The DNA_REPAIR pathway was only enriched in 395‐9B‐1 cells (Table 1). The common down‐regulated pathways in all three cell lines were COAGULATION and MYOGENESIS. The KRAS_SIGNALING_UP pathway was down‐regulated in 395‐7A‐1 and 395‐9B‐1 cells, whereas the INFLAMMATORY_RESPONSE pathway was down‐regulated in 395‐7A‐1 and 395‐6B‐1 cells (Table 1).

**Table 1:** The GSEA reports for pathways regulated in three immortalized cells compared with their parental G5DKO cells.

Regulation	395‐7A‐1	395‐6B‐1	395‐9B‐1
Up	G2M_CHECKPOINT	E2F_TARGETS	G2M_CHECKPOINT
	E2F_TARGETS	MTORC1_SIGNALING	E2F_TARGETS
	MYC_TARGETS_V1	TNFA_SIGNALING_VIA_NFKB	DNA_REPAIR
	MYC_TARGETS_V2	ESTROGEN_RESPONSE_EARLY	MTORC1_SIGNALING
	TNFA_SIGNALING_VIA_NFKB	BILE_ACID_METABOLISM	MYC_TARGETS_V1
	UNFOLDED_PROTEIN_RESPONSE	G2M_CHECKPOINT	PI3K_AKT_MTOR_SIGNALING
Down	ANGIOGENESIS	EPITHELIAL_MESENCHYMAL_TRANSITION	XENOBIOTIC_METABOLISM
	KRAS_SIGNALING_UP	ALLOGRAFT_REJECTION	EPITHELIAL_MESENCHYMAL_TRANSITION
	GLYCOLYSIS	KRAS_SIGNALING_UP	COAGULATION
	COAGULATION	INFLAMMATORY_RESPONSE	P53_PATHWAY
	MYOGENESIS	MYOGENESIS	MYOGENESIS
	INFLAMMATORY_RESPONSE	COAGULATION	ESTROGEN_RESPONSE_LATE

These results revealed that both growth signaling (E2F_TARGETS, MYC_TARGETS) and surveillance mechanism (G2M_CHECKPOINT, MITOTIC_SPINDLE) were up‐regulated, which might be essential for non‐tumorigenic immortalization. As we expected, the p53 pathway was down‐regulated in 395‐9B‐1 cell line because it carries the p53N236S mutation. Unexpectedly, the p53 pathway was also found to be down‐regulated in both 395‐7A‐1 and 395‐6B‐1 cells, although the p53 gene maintained WT.

In general, the ssGSEA analysis data are consistent with the GSEA results, whereas the ssGSEA data provided a way to compare between samples and reveal the gradual change pattern among samples.

### The genes regulated by immortalized cell lines derived from senescent WS MEFs

After obtaining an understanding of the pathways involved in immortalization escaped from senescence in these cells, we attempted to dissect the genes involved in this process. By using log_2_(fold change) = ±1 as the cutoff value, we obtained the gene lists for three immortalized cell lines with at an least two‐fold change in mRNA level compared to their parental G5DKO MEFs. venny, version 2.1.0 [13] was used to compare the common or unique differential expressed genes in 395‐7A‐1, 395‐6B‐1 and 395‐9B‐1 cells. The results showed that these three cell lines shared 182 (7.8%) common up‐regulated genes during immortalization, whereas most of the genes were unique to each cell line; for example, there were 599 genes (25.6%) uniquely up‐regulated in 395‐7A‐1 cells. On the other hand, for genes down‐regulated during immortalization, most of them (1069 genes; 27.6%) are common in these three cell lines. We then extracted these common or unique gene groups and aligned them with gene sets through the analysis function of the GSEA website. Interestingly, even with only 182 genes (7.8%), 57 out of 182 genes aligned with the DREAM_TARGETS pathway (Table 2), suggesting that up‐regulation of the DREAM_TARGETS pathway is essential for immortalization. Among those common genes, we noted Timeless, Cdt1, Mcm genes, Cenp genes, Birc5, Recql4, Pole, Foxm1, Cdkn1b, Cdkn2c, Sgo1, etc. These genes are known to be involved in either anti‐aging or quality control of the cell cycle. When we investigated the unique genes up‐regulated in each cell lines. The top1 gene set that aligned with genes uniquely expressed in 395‐7A‐1 cells was again the DREAM_TARGETS pathway, suggesting that this pathway played an essential role in 395‐7A‐1 cells (Table 2). We found this same pathway in uniquely expressed genes in 395‐9B‐1 cells, but not in uniquely expressed genes in 395‐6B‐1 cells. Instead, we found that 395‐7A‐1 and 395‐6B‐1 cells shared the NASOPHARYNGEAL_CARCINOMA_UP pathway in their uniquely expressed genes (Table 2). Surprisingly, the top 1 common pathway OLIGODENDROCYTE_DIFFERENTIATION_UP in all three cell lines was also found in uniquely expressed genes in 395‐9B‐1 cells (Table 2). OLIGODENDROCYTE_DIFFERENTIATION_UP is the gene set induced in the murine oligodendroglial precursor cell line Oli‐neu treated by an ErbB‐family kinase inhibitor [14]. Other than this, the key genes involved in germ cell production, such as PSMC3IP, are also up‐regulated in all three cell lines.

**Table 2:** The top pathways analyzed with the genes commonly (common) or uniquely (7A‐1, 6B‐1 and 9B‐1, respectively) up‐regulated in three immortalized cells (*P* < 0.00001).

Common	7A‐1	6B‐1	9B‐1
GOBERT OLIGODENDROCYTE DIFFERENTIATION UP	FISCHER DREAM TARGETS	DODD NASOPHARYNGEAL CARCINOMA UP	GOBERT OLIGODENDROCYTE DIFFERENTIATION UP
FISCHER DREAM TARGETS	DODD NASOPHARYNGEAL CARCINOMA DN	LEE BMP2 TARGETS UP	FISCHER DREAM TARGETS
DUTERTRE ESTRADIOL RESPONSE 24HR_UP	MEBARKI HCC PROGENITOR FZD8CRD UP	GRAESSMANN APOPTOSIS BY DOXORUBICIN DN	BERENJENO TRANSFORMED BY RHOA UP
MARSON BOUND BY E2F4 UNSTIMULATED	JOHNSTONE PARVB TARGETS 3 DN	WEST ADRENOCORTICAL TUMOR DN	CHEN METABOLIC SYNDROM NETWORK
KOBAYASHI EGFR SIGNALING 24HR DN	GOBERT OLIGODENDROCYTE DIFFERENTIATION UP	WANG MLL TARGETS	PUJANA BRCA1 PCC NETWORK
BENPORATH CYCLING GENES	KINSEY TARGETS OF EWSR1 FLII USION UP	BERENJENO TRANSFORMED BY RHOA DN	NUYTTEN EZH2 TARGETS DN

We then analyzed the genes with a two‐fold change that were down‐regulated by immortalization. Among the genes that were commonly down‐regulated in all three cell lines, the top1 aligned gene set is NABA_MATRISOME, which is the ensemble of genes encoding extracellular matrix and extracellular matrix‐associated proteins. The other top two gene sets, MEISSNER_BRAIN_HCP_WITH_H3K4ME3_AND_H3K27ME3 and BENPORATH_SUZ12_TARGETS, were related to epigenetic regulation (Table 3). Among those genes, we found CXCL14, SPON1, WNT genes, BMP genes, ADAM metallopeptidase, MMP genes, IGF1, etc.

**Table 3:** The top pathways analyzed with the genes commonly (common) or uniquely (7A‐1, 6B‐1 and 9B‐1, respectively) down‐regulated in three immortalized cells (*P* < 0.00001).

Common	7A‐1	6B‐1	9B‐1
NABA MATRISOME	CHEN METABOLIC SYNDROM NETWORK	HAMAI APOPTOSIS VIA TRAIL UP	DURAND STROMA S UP
MEISSNER BRAIN HCP WITH H3K4ME3 AND H3K27ME3	LEE BMP2 TARGETS UP	DACOSTA UV RESPONSE VIA ERCC3 DN	GRAESSMANN APOPTOSIS BY DOXORUBICIN UP
BENPORATH SUZ12 TARGETS	REN ALVEOLAR RHABDOMYOSARCOMA DN	MILI PSEUDOPODIA HAPTOTAXIS UP	GRAESSMANN RESPONSE TO MC AND DOXORUBICIN UP
BENPORATH EED TARGETS	BERENJENO TRANSFORMED BY RHOA DN	RODRIGUES THYROID CARCINOMA ANAPLASTIC UP	QUINTENS EMBRYONIC BRAIN RESPONSE TO IR
BENPORATH ES WITH H3K27ME3	MILI PSEUDOPODIA HAPTOTAXIS DN	CUI TCF21 TARGETS 2 DN	WANG MLL TARGETS
LEE BMP2 TARGETS UP	GOBERT OLIGODENDROCYTE DIFFERENTIATION DN	GABRIELY MIR21 TARGETS	LEE BMP2 TARGETS UP

### A comparison of G2M and DREAM pathway related genes in three immortalized cell lines

The above results showed that the gene expression profile in three immortalized cell lines was enriched in G2M and DREAM pathways. As we know, these two gene sets are partially overlapped and functionally highly related. To further analyze the genes involved in the biological function of these two pathways, as well as dissect the genes commonly or uniquely expressed in each cell line, we further analyzed the genes enriched for the G2M and DREAM pathways from GSEA reports.

**Fig. 2:**
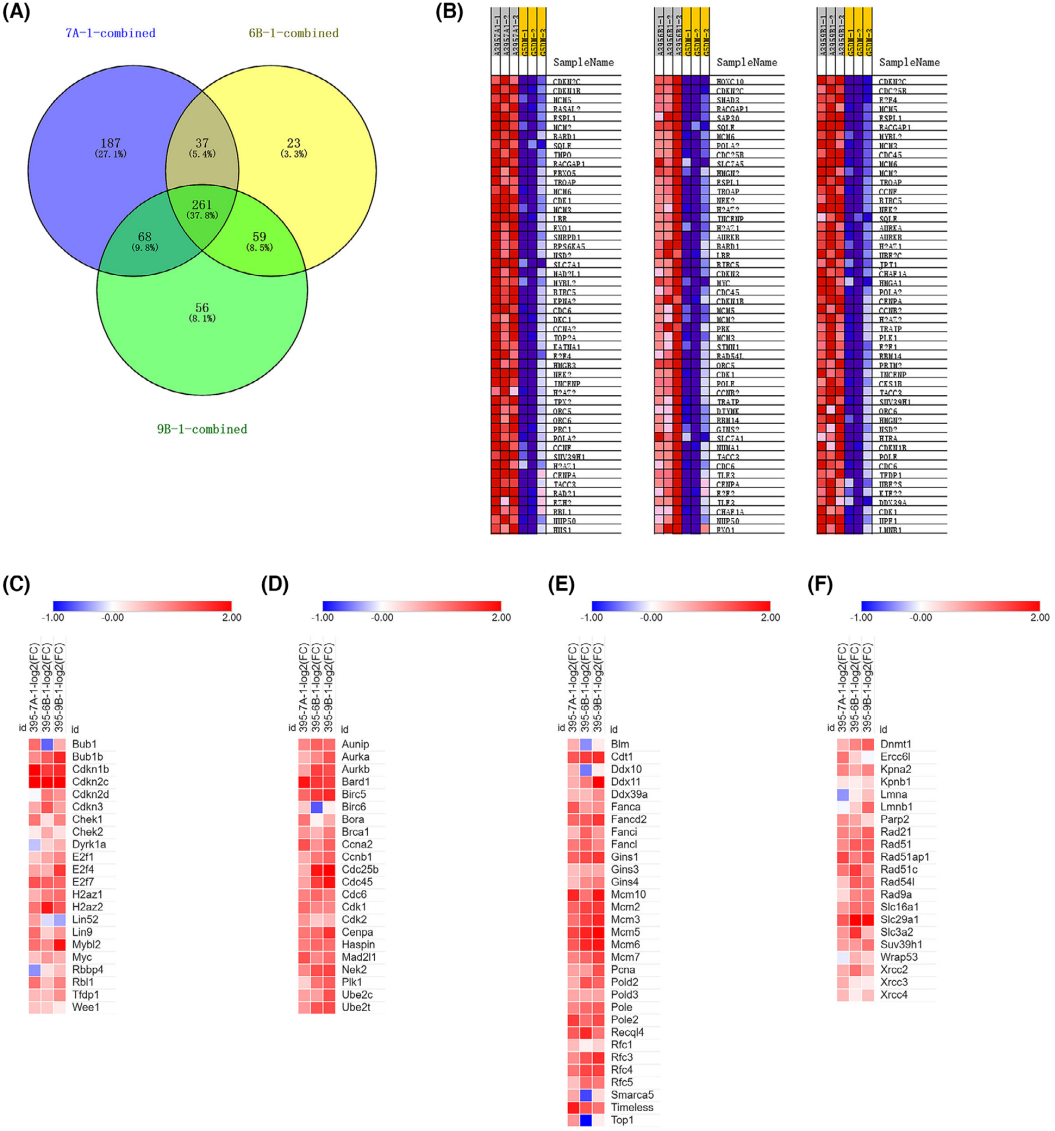
Analysis of individual genes contributing to the up‐regulation of DREAM and G2M checkpoint pathway in 395‐7A‐1, 395‐6B‐1 and 395‐9B‐1 cells compared to their parental G5DKO cells. (A) Venn diagram of common or unique genes in 395‐7A‐1, 395‐6B‐1 and 395‐9B‐1 cells, which contributed to the up‐regulation of the DREAM and G2M checkpoint pathway. The 395‐7A‐1, 395‐6B‐1 and 395‐9B‐1 cells share 37.8% commonly up‐regulated genes in the DREAM and G2M checkpoint pathway. (B) Heatmap of the top 50 genes contributing to the up‐regulation of G2M checkpoint pathway in 395‐7A‐1, 395‐6B‐1 and 395‐9B‐1 cells according to GSEA score. (C) Heatmap of the differential regulation of DREAM pathway and cell cycle inhibiting related genes in 395‐7A‐1, 395‐6B‐1 and 395‐9B‐1 cells according to the value of log_2_(fold change). (D) Heatmap of the differential regulation of cell cycle and mitotic progression related genes in 395‐7A‐1, 395‐6B‐1 and 395‐9B‐1 cells according to the value of log_2_(fold change). (E) Heatmap of the differential regulation of DNA helicase and DNA replication related genes in 395‐7A‐1, 395‐6B‐1 and 395‐9B‐1 cells according to the value of log_2_(fold change). (F) Heatmap of the differential regulation of DNA repair, epigenetic modification, metabolic pathway and nuclear membrane function related genes in 395‐7A‐1, 395‐6B‐1 and 395‐9B‐1 cells according to the value of log_2_(fold change).

For each cell line, we combined the up‐regulated genes that contributed to the G2M and DREAM pathways according to the significance in GSEA report, and deleted the duplicate genes, generating the up‐regulated gene list in the DREAM/G2M pathways. Again, using venny, version 2.1.0 [13], we compared the common and unique differential expressed DREAM/G2M genes in 395‐7A‐1, 395‐6B‐1 and 395‐9B‐1 cells. The results showed that 261 genes (37.8%) were commonly up‐regulated in all three cell lines (Fig. 2A), suggesting that those genes played an essential role in the immortalization status. Interestingly, 395‐7A‐1 cells had an extra unique 187 genes (27.1%) up‐regulated in the DREAM/G2M pathways, which might contribute to its unique non‐tumorigenic characteristics. The heatmap for the top 50 genes that contributed to the G2M checkpoint pathway in each cell line is shown in Fig. 2B. Interestingly, we found that both cell cycle inhibiting factor Cdkn2c and DNA replication promoting factor Mcm were present in the top 10 genes (Fig. 2B). At the same time, the helicase family genes were up‐regulated, suggesting promotion of the cell cycle, although with a better quality control mechanism. The full heatmaps for genes contributing to the G2M_CHECKPOINT and DREAM_TARGETS pathways in these three cell lines are shown in Figs S1 and S2.

To further compare the differential regulation of those genes among these three cell lines, we choose key genes from the DREAM/G2M pathways and used their value of log_2_(fold change) to plot a heatmap. We found that both suppression and activation forms of DREAM factors for G2M progression are up‐regulated in these immortalized cells (Fig. 2C). Interestingly, both the suppressive factors TFDP1 (DP1), E2F4, H2AZ1, H2AZ2 and Rbl1 (p107) and the activation factors Mybl2 and E2F1 were up‐regulated in all three cell lines. Other than this, the MuvB core components Lin9 and Lin52 were also up‐regulated in 395‐7A‐1 cells, and Rbbp4 and Dyrk1a were up‐regulated in 395‐9B‐1 cells, suggesting that enhanced DREAM regulation occurred in all three cell lines (Fig. 2C). Again, we found that the cell cycle inhibitors Cdkn2c, Cdkn1b and Bub1b were up‐regulated in all three cell lines, whereas Cdkn2c was up‐regulated in 395‐6B‐1 and 395‐9B‐1 cells, and Cdkn3 was up‐regulated in 395‐6B‐1 cells (Fig. 2C). Also, the cell cycle negative regulators Bub1, Wee1 and Chek1 were up‐regulated in 395‐7A‐1 cells, whereas Chek2 was up‐regulated in 395‐6B‐1 cells (Fig. 2C). These data suggested that the cell cycle progression is under tighter control in these immortalized cells through the DREAM pathway.

On the other hand, the cell cycle promoting factors Ccna2, Cdc6, cdk1, Ccnb1, Cdc25b and Cdc45 were up‐regulated in all three cell lines (Fig. 2D). The mitotic spindle related genes Aurka, Aurkb, Aunip, Cenpa, Mad2L1 and Haspin were also up‐regulated in all three cell lines (Fig. 2D). DNA replication or helicase related genes Mcm family genes, Fanconi family genes, polymerase genes, Rfc genes, Gins genes, Ddx genes, Timeless, Cdt1, Blm, Top1 and Recql4 were all up‐regulated in all three cell lines (Fig. 2E). Together, these data suggested the enhancement of DNA replication regulation and the G2M checkpoint, which are known to be the downstream pathways of DREAM.

Importantly, we found that the DNA repair related genes Rad9a, Rad51, Rad54l, Rad21, Rad51c, Rad51ap1, Xrcc2, Xrcc3, Xrcc4, Ercc6l and Parp2 were up‐regulated to different extents in all three cell lines (Fig. 2F), suggesting the up‐regulation of DNA repair ability. It is worth noting that the nuclear membrane function related gene Kpna2 was up‐regulated in all three cell lines, and Lmnb1 was up‐regulated in 395‐9B‐1 cells, whereas Kpnb1 and Lmna were mildly up‐regulated in 395‐9B‐1 cells, suggesting the up‐regulation of nuclear membrane function (Fig. 2F). The histone methyltransferase Suv39h1 and the DNA methyltransferase Dnmt1 were up‐regulated to different extents in all three cell lines, suggesting a switch of chromatin status (Fig. 2F). The proton‐linked monocarboxylate transporters Slc16a1, Slc3a2 and Slc29a1 were up‐regulated to different extents in all three cell lines, suggesting an active metabolism status in immortalized cells (Fig. 2F). Wrap53, which down‐regulates p53 function, was found to be up‐regulated in 395‐6B‐1 cells (Fig. 2F).

**Fig. 3:**
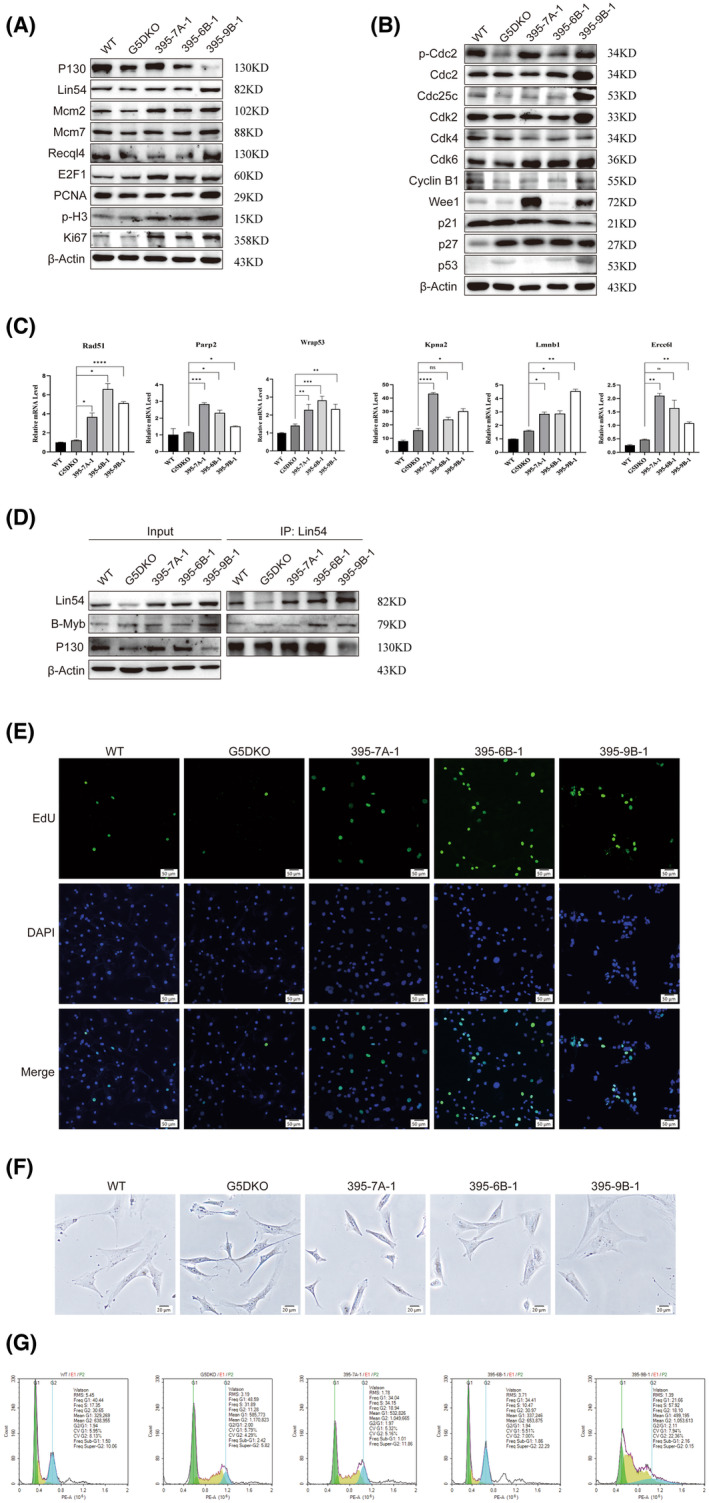
The DREAM/G2M pathway and cell cycle related proteins were regulated to balance the tumor inhibitory and cell cycle re‐entry in immortalized cells. (A) The DREAM/G2M pathway related proteins were regulated in immortalized cells, which might contribute to the tumor inhibitory mechanism, as well as cell cycle progression in those non‐tumorigenic cells. The phosphorylated H3 (p‐H3) and Ki67 proteins were used as the proliferation markers. (B) The cell cycle related proteins were up‐regulated in immortalized cells compared to G5DKO cells, which might contribute to the re‐entry of cell cycle. (C) By quantitative PCR analysis, the DNA repair related genes were revealed to be up‐regulated in immortalized cells compared to G5DKO cells. The data were analyzed using prism, version 9; http://www.graphpad‐prism.cn) by one‐way analysis of variance. All experiments were repeated at least three times with three replicates each time. The error bars indicate the SD. Statistical significance is indicated with asterisks: *P < 0.05; **P < 0.01; ***P < 0.001; ****P < 0.0001; ns, no significance. (D) The co‐immunoprecipitation data revealed that both activation (B‐Myb) and inhibitory (p130) form of DREAM proteins were up‐regulated and bound to the core protein Lin54. (E) EdU incorporation revealed robust cell proliferation in immortalized cells compared to G5DKO cells. Scale bar = 50 μm. (F) Using SA‐β‐Gal staining, the G5DKO cells manifested more obvious SA‐β‐Gal positive cells than 395‐7A‐1, 395‐6B‐1 and 395‐9B‐1 cells. Scale bar = 20 μm. (G) A flow cytometry assay indicated a reduced percentage of G1 phase and an increased percentage of G2 phase in 395‐7A‐1, 395‐6B‐1 and 395‐9B‐1 cells.

To further confirm activation of the DREAM/G2M pathway in these cells, we detected the related protein level in 395‐7A‐1, 395‐6B‐1 and 395‐9B‐1 cells, with WT and G5DKO cells as controls. The data showed that the cell cycle suppression factor Rbl2 (p130) was up‐regulated dramatically in 395‐7A‐1 cells compared to its parental cell G5DKO, remained unchanged in 395‐6B‐1 cells and was down‐regulated in 395‐9B‐1 cells (Fig. 3A). The WT cells showed a relatively high level of p130 expression. Lin54, the core factor for both cell cycle suppression and the activation form of DREAM, was up‐regulated in both 395‐7A‐1 and 395‐9B‐1 cells compared to their parental G5DKO cells (Fig. 3A). Interestingly, DNA helicases Mcm2 and Mcm7, which are known to be downstream effectors of the DREAM pathway, were up‐regulated in 395‐7A‐1, 395‐6B‐1 and 395‐9B‐1 cells (Fig. 3A). The telomere function related DNA helicase Recql4 was up‐regulated 395‐9B‐1 cells, but not much changed in 395‐7A‐1 and 395‐6B‐1 cells (Fig. 3A). The cell cycle promoter E2F1 was also up‐regulated in 395‐7A‐1, 395‐6B‐1 and 395‐9B‐1 cells (Fig. 3A), whereas DNA replication factor PCNA was only obviously up‐regulated in 395‐9B‐1 cells (Fig. 3A). Interestingly, the cell proliferation markers p‐H3 and Ki67 were both up‐regulated in 395‐7A‐1, 395‐6B‐1 and 395‐9B‐1 cells (Fig. 3A), indicating that regulation was beneficial for cell proliferation. These data confirmed the RNA‐seq data, and suggested that both suppression and activation form of DREAM factors are up‐regulated in these immortalized cells, which might contribute to the balance of cell growth and tumor inhibition.

We further detected the expression level of cell cycle regulators in those immortalized cells. The data showed that the phosphorylated Cdc2 (p‐Cdc2) was up‐regulated in 395‐7A‐1, 395‐6B‐1 and 395‐9B‐1 cells compared to G5DKO, whereas Cdc2, Cdc25c and Cdk2 were up‐regulated only in 395‐9B‐1 cells (Fig. 3B). The Cdk4 and Cyclin B1 level did not change much among the cells, whereas Cdk6 was up‐regulated in 395‐7A‐1, 395‐6B‐1 and 395‐9B‐1 cells compared to G5DKO and WT cells (Fig. 3B). Interestingly, we found that cell cycle inhibitor Wee1 was also up‐regulated in 395‐7A‐1 and 395‐9B‐1 cells compared to parental G5DKO cells (Fig. 3B). The cell cycle inhibitor p21 was down‐regulated in 395‐7A‐1, 395‐6B‐1 and 395‐9B‐1 cells and p27 was not changed compared to parental G5DKO cells (Fig. 3B).

We also used a quantitative PCR to verify the differential expression of DREAM down‐stream DNA repair related genes in these immortalized cells. The results showed that, compared with their parental G5DKO cells, the genes Rad51, Wrap53, Parp2, Lmnb1, Ercc6l and Kpna2 were all up‐regulated in 395‐7A‐1, 395‐6B‐1 and 395‐9B‐1 cells (Fig. 3C). These data further confirmed the RNA‐seq data and suggested the enhancement of DNA repair capacity in 395‐7A‐1, 395‐6B‐1 and 395‐9B‐1 cells.

The key regulating by DREAM core protein (such as Lin54 and Lin52) is to bind to different proteins and form different forms of the DREAM complex accordingly, which activate or inhibit cell cycle progression. To compare the differential binding partners before and after immortalization, we used a co‐immunoprecipitation technique for those cells. Using Lin54 (the common core protein for both DREAM forms) antibody for pull down, we confirmed that Lin54 abundance was up‐regulated in 395‐7A‐1, 395‐6B‐1 and 395‐9B‐1 cells (Fig. 3D). For the proteins bound to Lin54, we found more p130 (the key protein for DREAM inhibitory form) bound to Lin54 in 395‐7A‐1 and 395‐6B‐1 compared to G5DKO cells, whereas less p130 bound to Lin54 in 395‐9B‐1 cells (Fig. 3D). On the other hand, the data showed that more B‐Myb (the key protein of activation form) bound to Lin54 in 395‐6B‐1 and 395‐9B‐1 compared to G5DKO cells, with not much change in 395‐7A‐1 cells (Fig. 3D). These binding patterns matched very well with the cell growth and tumorigenesis capacity, which is 395‐9B‐1 > 395‐6B‐1 > 395‐7A‐1 [10, 11].

To investigate the cellular consequence of this new balance of DREAM active form and inhibitory form, we used EdU incorporation to test cell proliferation. The data indicated that 395‐7A‐1, 395‐6B‐1 and 395‐9B‐1 cells showed much more robust EdU incorporation than WT or G5DKO cells (Fig. 3E), suggesting an enhancement of cell proliferation. Using SA‐β‐Gal staining, we found that G5DKO cells manifested obvious SA‐β‐Gal positive cells (Fig. 3F, G5DKO), whereas not many SA‐β‐Gal positive cells were observed in 395‐7A‐1, 395‐6B‐1 and 395‐9B‐1 cells (Fig. 3F). By flow cytometry, we further analyzed the cell cycle progression of these cells. As we expected, compared with G5DKO cells, we observed a reduced percentage of G1 phase in 395‐7A‐1, 395‐6B‐1 and 395‐9B‐1 cells (Fig. 3G). Interestingly, we also observed an increased percentage of G2 phase in 395‐7A‐1, 395‐6B‐1 and 395‐9B‐1 cells. These data suggest a smoother G1 progress in these immortalized cells, and an enhanced check in G2/M progression.

Together, these data confirm previous RNA‐seq data and indicate that a complicated cell cycle regulating and monitoring network was regulated more tightly in non‐tumorigenic immortalized cells via the DREAM pathway.

## Discussion

It is of great interest to identify aging‐related genes and apply this knowledge to the interference of aging‐related diseases. Many studies have been conducted aiming to analyze the longevity related gene signatures and pathways, which provide databases for anti‐aging signatures. For the treatment of aging‐related diseases, we still need to identify the signatures that could rescue aging related phenotypes without tumor risk.

In the present study, we performed RNA‐seq and analysis of the transcription pattern for three non‐tumorigenic immortalized cell lines: 395‐7A‐1, 395‐6B‐1 and 395‐9B‐1. Using ssGSEA and GSEA assays, we identified the DREAM pathway, and its related G2M checkpoint, mitotic spindle, DNA helicase and replication as the essential pathways for maintaining non‐tumorigenic cell survival (Figs 1 and 3). The DREAM pathway is actually very complicated. It comprises integrated cell cycle regulating pathways such as TP53, DREAM, MMB‐FOXM1 and RB‐E2F, etc. [15, 16, 17, 18]. It is composed of both positive and negative regulators of the cell cycle, with some referring to the cell cycle suppressing complex as DREAM and the cell cycle promoting complex as MMB‐FOXM1 [19, 20]. We speculate that the enhancement of DREAM pathway might result in tighter and safer regulation of the cell cycle, which contributes to the escape from senescence and non‐tumorigenic immortalization. Interestingly, the uniquely up‐regulated genes in 395‐7A‐1 cells, the least tumorigenic among the three cell lines, also matched very well with the DREAM pathway (Table 2). These data further suggest that the DREAM pathway plays an essential dual role in balancing tumorigenesis and aging during the immortalization of senescent cells. Our data also revealed that the immortalized cells enabled an increase of cell proliferation and controlled cell cycle progression, indicating that regulation benefited cell proliferation (Fig. 3E–G). Given our previous finding of the key role of p21 in tumor suppression in these immortalized cells [11], we further revealed the important role of DREAM (p21 downstream pathway) in tumor suppression for these tumor‐free immortalization cells.

Importantly, in this pathway, we found that the DNA helicase and DNA replication related genes were up‐regulated, such as Mcm genes, Timeless, Gins genes, etc., which might enhance the quality of DNA replication and help improve telomere function via alternative lengthening of telomere [21]. Because the three immortalized cells used in the present study were telomerase negative cells, our data connected the DREAM pathway with telomere lengthening regulation, which is known to be essential for cell immortalization or organism longevity [22].

In this pathway, we found the genes involved in the DNA repair pathway, such as Rad, Ercc, Xrcc and Fanconi anemia genes. We also found metabolic related genes, such as monocarboxylate transporter Slc genes, as well as chromosome organization related genes, including Aurk genes, Cenp genes, Mad2l1, Haspin, Birc etc. (Fig. 2). These pathways have been identified as top pathways in longevity across different mammalian species [1].

On the other hand, the down‐regulation of epigenetic modification, extracellular matrix and K‐Ras signaling might be important to maintaining a low inflammation level. The senescence associated secretory phenotype includes proinflammatory molecules and extracellular matrix remodeling [23, 24]. Through transcriptomic profiles of endothelial replication‐induced senescence and senescence induced by inflammatory cytokines, it was found that multiple targets of p53/p16‐RB‐E2F‐DREAM were repressed in senescent cells [25]. Our data further support this connection between DREAM and inflammation. It has also been shown that tetraspanin CD53 promoted the activity of DREAM transcriptional repressor complex, which downregulated genes associated with cycling and protected inflammation stressed hematopoietic stem cells in quiescence [26].

In terms of the pathway pattern, 395‐7A‐1 and 395‐9B‐1 are similar, whereas 395‐6B‐1 and 395‐9B‐1 are similar in gene signatures. By comparing the immortalization related genes with a previous study [5], we could identify a few overlapped genes associated with longevity. This phenomenon was also reported in a previous study reporting that only markers at APOE and FOXO3A were well replicated in genetic associations with exceptional longevity [3]. However, with a gene set or pathway comparison, we could identify a common functional regulation in other studies, such as DNA repair ability, helicase activity, metabolic activity and inflammation response, etc. We speculate that gene set based pathway analysis is more compatible than comparison of individual genes.

The signature pathways and genes revealed in the present could be useful targets or readout for either clinical pharmacological studies or for drug screening for a safe anti‐aging strategy. The signature pathways might be more stable and reliable as biomarkers than individual genes for clinical practice.

## Materials and methods

### Cell culture

The three spontaneously immortalized MEF cell lines, 395‐7A‐1, 395‐6B‐1 and 395‐9B‐1, were gifts from Sandy Chang from Yale University (New Haven, CT, USA). The 395‐7A‐1, 395‐6B‐1 and 395‐9B‐1 cells were independently immortalized from senescent WS MEFs as previously described [8, 10]. The number 395 stands for the code of the parental WS MEF, and 7A, 6B or 9B stand for the code of different culture dishes, followed by the immortalized clone number 1. The primary and immortalized MEF cells were cultured in Dulbecco's modified Eagle's medium with 10% fetal bovine serum at 37 °C with 5% CO_2_ and 3% O_2_. The WT MEFs were used for experiments before passage 5.

### RNA‐seq and GSEA

Cell samples were collected and sent for commercial RNA sequencing service (Novogene, Beijing, China). Briefly, the total RNA was extracted and enriched by oligo‐dT labeled magnetic beads, and used to construct a library for RNA‐seq. The sequenced reads (raw reads) were evaluated for quality control. The adapters and low quality reads were filtered to obtain clean reads. The clean data were then aligned with the reference mouse genome using tophat2(https://ccb.jhu.edu/software/tophat/index.shtml). The RNA counts were annotated and the FPKM (i.e. fragments per kilobase of sequence per million mapped reads) file was generated for bioinformatics analysis. The Bioinformatics ExperT SYstem (BETSY) was applied to automate the development of workflows [27]. ssGSEA was applied to analyze the RNA‐seq data. Hallmark (designed for well‐defined biological states and processes) gene sets [28] from the Molecular Signatures Database were used for ssGSEA analysis. The heatmaps were plotted with BETSY by centering with mean but without hierarchical clustering.

To calculate fold change, we used r, version 3.5.1 (Institute for Statistics and Mathematics, Vienna, Austria; https://www.r‐project.org) to perform edge R analysis and normalize the RNA‐seq reads. The normalized RNA‐seq reads were also applied to gsea, version 4.1.0 (Massachusetts Institute of Technology, and Regents of the University of California; http://www.gsea‐msigdb.org/gsea/downloads.jsp) to perform GSEA.

### Gene expression signature analysis

venny, version 2.1.0 [13] was used to compare the common and unique differential expressed genes in 395‐7A‐1, 395‐6B‐1 and 395‐9B‐1 cells and generate the common or unique up‐regulated or down‐regulated gene lists. The gene lists were then applied to the Molecular Signatures Database, version 7.4, to compute overlaps between our gene lists and gene sets in MSigDB.

The heatmap was plotted either in gsea or using morpheus (Versatile matrix visualization and analysis software, https://software.broadinstitute.org/morpheus/).

### Western blotting

Cells were harvested and lysed in RIPA buffer containing protease inhibitor cocktail (Roche, Basel, Switzerland). The 20 μg of total protein was separated by SDS/PAGE and then transferred to a poly(vinylidene difluoride) membrane. After blocking in 10% non‐fat milk for 1 h at room temperature, membranes were incubated with primary antibodies overnight at 4 °C. The membranes were then incubated with horseradish peroxidase‐labeled secondary antibodies, and visualized with electrochemiluminescence. The primary antibodies used were anti‐Mcm7 (dilution 1 : 1000; Santa Cruz Biotechnology, Santa Cruz, CA, USA), anti‐Mcm2 (dilution 1 : 1000; Abcam, Cambridge, UK), anti‐Recql4 (dilution 1 : 1000; Invitrogen, Waltham, MA, USA), anti‐p130 (dilution 1 : 1000; Abcam), anti‐Wee1 (dilution 1 : 1000; Abcam), anti‐Lin54 (dilution 1 : 1000; Abcam), anti‐E2F1 (dilution 1 : 1000; Novus, St Louis, MO, USA), anti‐b‐Myb (dilution 1 : 1000; Abmart, Shanghai, China), anti‐Cyclin B1 (dilution 1 : 2000; CST, Danvers, MA, USA), anti‐Cdc2 (dilution 1 : 1000; CST), anti‐Cdc25C (dilution 1 : 1000; SAB, Greenbelt, MD, USA), anti‐phospho‐Cdc2 (Tyr15) (dilution 1 : 1000; CST), anti‐Cdk2 (dilution 1 : 1000; CST), anti‐Cdk4 (dilution 1 : 1000; Santa Cruz Biotechnology), anti‐Cdk6 (dilution 1 : 2000; CST), anti‐PCNA (dilution 1 : 1000; Abcam), anti‐p21 (dilution 1 : 500; Santa Cruz Biotechnology), anti‐p27 (dilution 1 : 3000; BD, Franklin, Lakes, NJ, USA), anti‐p53 (dilution 1 : 3000; Proteintech, St Leon‐Rot, Germany), anti‐phosphorylated H3 (p‐S10, dilution 1 : 1000; Abcam), anti‐Ki67 (dilution 1 : 1000; CST), anti‐actin (dilution 1 : 1000; Santa Cruz Biotechnology).

### Co‐immunoprecipitation

The cells were harvested and cell lysates were prepared using mild RIPA buffer (Beyotime, Shanghai, China) with proteinase inhibitor cocktail (Roche). The protein concentration of cell lysates was measured and adjusted to same concentration. The cell lysates were precleared with Protein A/G Magnetic Beads (Thermo Fisher Scientific, Waltham, MA, USA) at 4 °C for 2 h. Then, the supernatants were collected and incubated with the same Protein A/G Magnetic Beads and primary antibodies at 4 °C overnight. The precipitates were collected and used for Western blotting analysis. The primary antibody used was anti‐Lin9 (dilution 1 : 100; Proteintech).

### Quantitative PCR

The RNA from 395‐7A‐1, 395‐6B‐1 and 395‐9B‐1 cells was isolated using Trizol followed by purification with the RNeasy Mini kit (Qiagen, Hilden, Germany). The cDNA was reverse transcribed and the quantitative PCR was performed on an ABI Prism 7300 sequence detection system with SYBR‐Green PCR master mix in accordance with the manufacturer's instructions (Applied Biosystems, Waltham, MA, USA). The primers used were: Rad51‐F: CCAGACCCAGCTCCTTTACC, Rad51‐R: CACTGCGACACCAAACTCATC; Wrap53‐F: GGAATCGAGGAGCAAGATGTTT, Wrap53‐R: GAGAAGCTGTAGGTCCAAAAGG; Parp2‐F: GCAACAGAAGACGACTCTCCT, Parp2‐R: CAGCCATAGGCCCTTTTCTCT; Lmnb1‐F: GAGTATGAGGCGGCACTAAAC, Lmnb1‐R: CATCTGCTAACTGCTTTTTGGC; Ercc6l‐F: TCTCCCTTTCCATTCTCATCTGTG, Ercc6l‐R: CCTCCTGTATCTTCCCGCACTC; Kpna2‐F: ATGTCCACGAACGAGAATGCT, Kpna2‐R: AAGGAGCTGACGTTTCTTCTTTT.

### EdU incorporation and SA‐β‐Gal staining

For EdU incorporation, the cells were cultured 2 h with 10 μm EdU, followed by fixation with 4% paraformaldehyde and permeabilization with 0.3% Triton X‐100. A BeyoClick EdU‐488 cell proliferation kit (Beyotime) was used to perform the Click reaction and stain the incorporated EdU. SA‐β‐Gal staining was performed as described previously [29]. Briefly, cultured cells were fixed and stained for SA‐β‐galactosidase activity at 37 °C for 4 h.

## Conflicts of interest

The authors declare that they have no conflicts of interest.

### Peer review

The peer review history for this article is available at https://www.webofscience.com/api/gateway/wos/peer‐review/10.1002/2211‐5463.13748.

## Author contributions

LJ and JT performed the western blotting and co‐immunoprecipitation. HL and JD prepared the RNA‐seq samples and performed the transcriptome analysis. YL designed the study and wrote the manuscript.

## Supporting information


**Fig. S1.** Heatmap of genes contributing to the up‐regulation of G2M checkpoint pathway in 395‐7A‐1, 395‐6B‐1 and 395‐9B‐1 cells according to GSEA score.

**Fig. S2.** Heatmap of genes contributing to the up‐regulation of DREAM pathway in 395‐7A‐1, 395‐6B‐1 and 395‐9B‐1 cells according to GSEA score.

**Table S1.** GSEA results summary.

## Data Availability

The datasets and the materials used in the present study are available from the corresponding author on reasonable request.
