# Supplementary figures and images for: The dual role of the DREAM/G2M pathway in non‐tumorigenic immortalization of senescent cells
Source: FEBS Open Bio. 2023 Dec 21;14(2):331–43. doi: 10.1002/2211-5463.13748 (PMC10839291; doi:10.1002/2211-5463.13748)

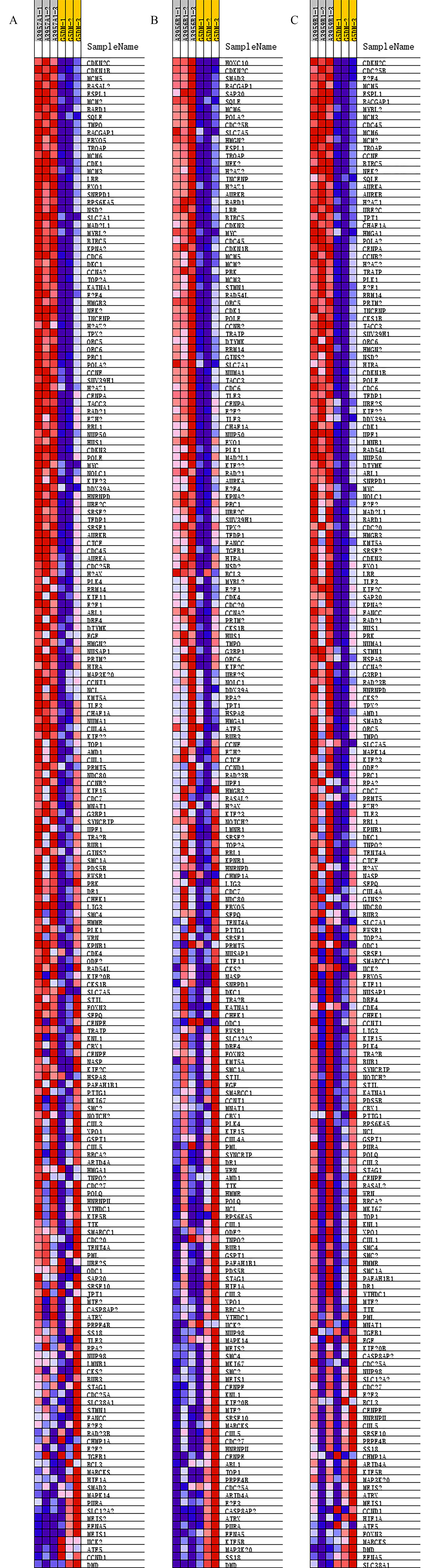

Supplement: Supplementary file 1 — Fig. S1. Heatmap of genes contributing to the up‐regulation of G2M checkpoint pathway in 395‐7A‐1, 395‐6B‐1 and 395‐9B‐1 cells according to GSEA score. [file FEB4-14-331-s001.jpg]
